# Supplementary material for: Choice-induced preference change under a sequential sampling model framework
Source: Sci Rep. 2026 Mar 21;16:14455. doi: 10.1038/s41598-026-44610-5 (PMC13149806; doi:10.1038/s41598-026-44610-5)
Supplement: Supplementary file 1 — Supplementary Information. [file 41598_2026_44610_MOESM1_ESM.docx]

**Supplementary Material**

**for**

**Choice-Induced Preference Change under a Sequential Sampling Model Framework**

Douglas G. Lee ^1,*^ Giovanni Pezzulo ^1^

^1^ Institute of Cognitive Sciences and Technologies, National Research Council

Via Giandomenico Romagnosi 18a, 00196 Roma, Italy

*Relationships between model parameters and SoA*

In this study, we demonstrated that sequential-sampling models such as the drift-diffusion model (DDM) can generate spreading of alternatives (SoA) between choice options under a reasonable set of assumptions. Including information about individual attributes can improve model performance in terms of matching empirical data patterns. Beyond this, we thought it might be interesting or useful to further delve into which factors (model parameters) most affect SoA under the formulations we considered. Figure S1 illustrates the relationship between each model parameter (across the model variants) and the simulated levels of SoA (mean per participant). The response threshold had a non-monotonic relationship, where low or high threshold heights led to lower mean SoA and mid-range threshold heights led to higher mean SoA. The drift rate(s) had negative relationships, with very low drift rates having the highest mean SoA. The relationship with non-decision time was flat for low- and mid-range values, but negative for high-range values. Finally, the start times for pleasure and nutrition had different relationships. However, the more important measure is the difference in start times (relative start time of nutrition versus pleasure). This measure had a similar relationship as non-decision time — mostly flat with a downturn for high values.


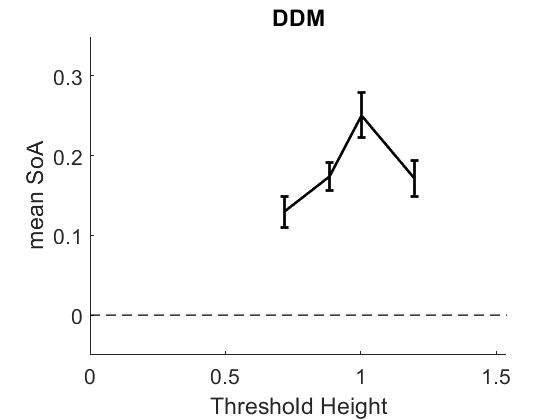

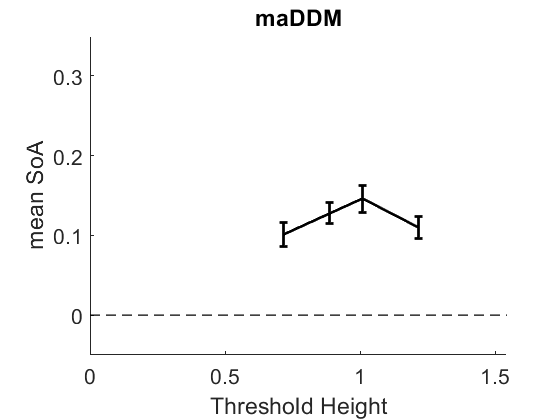

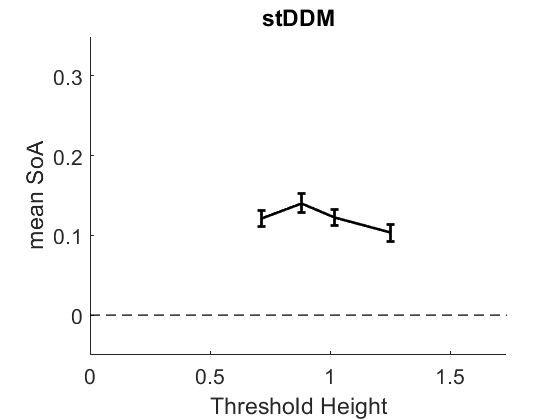


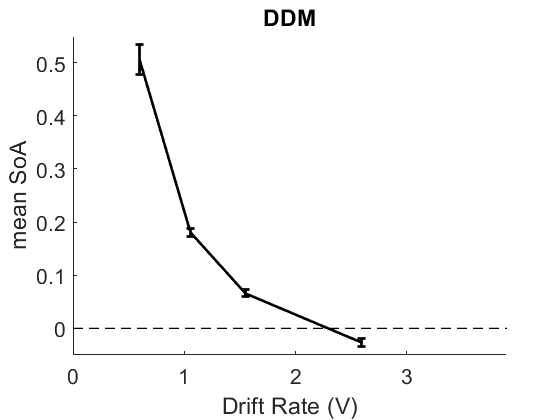


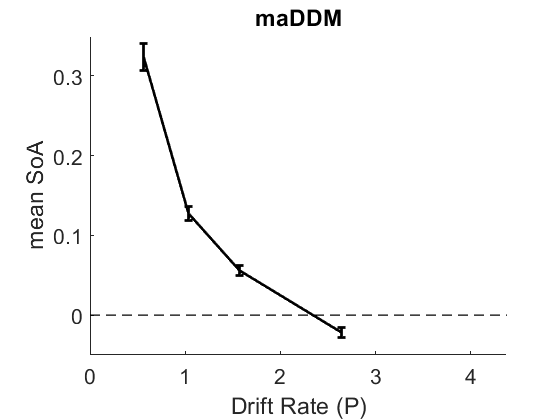

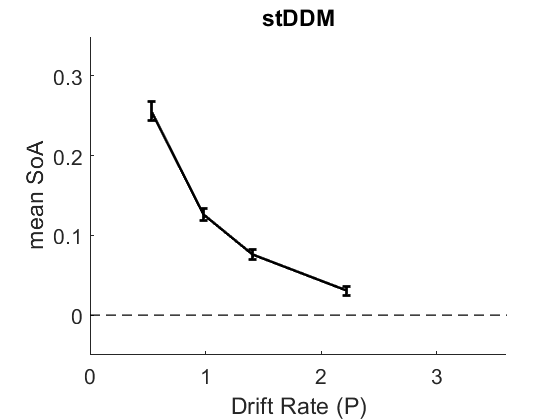


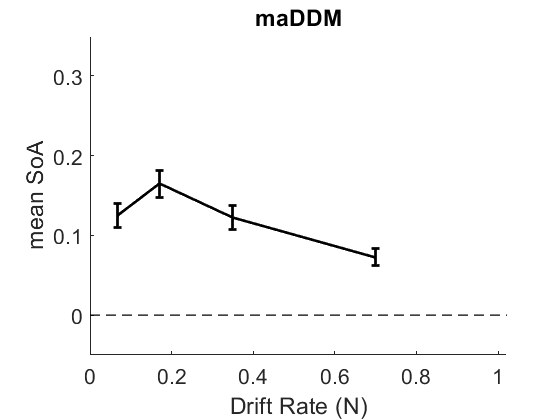

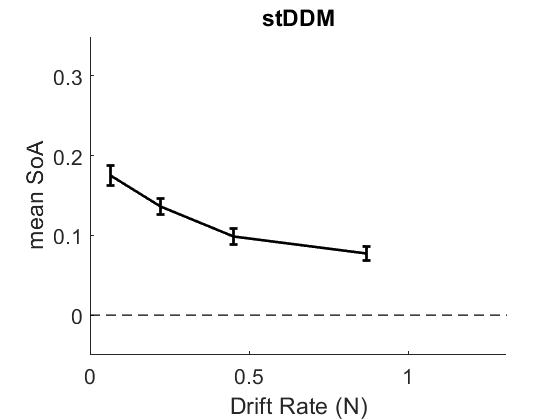


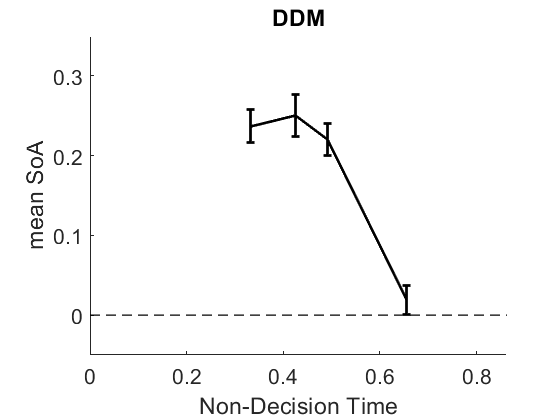

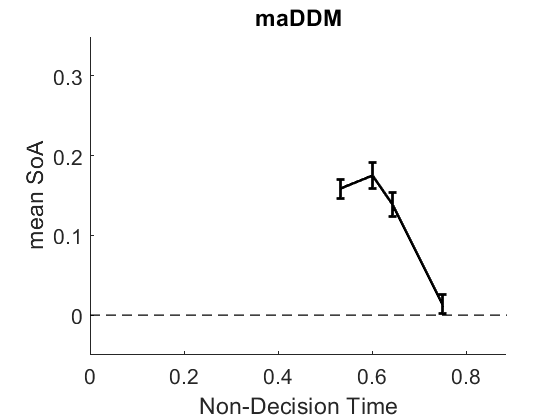

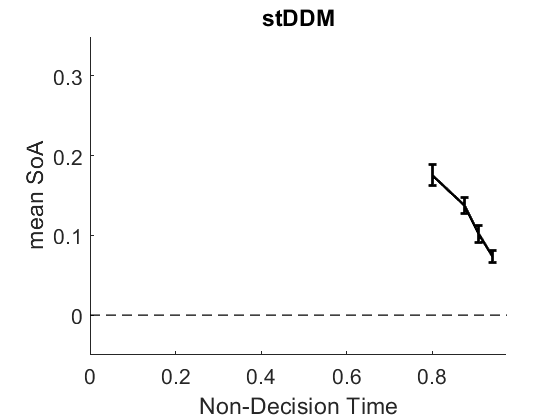


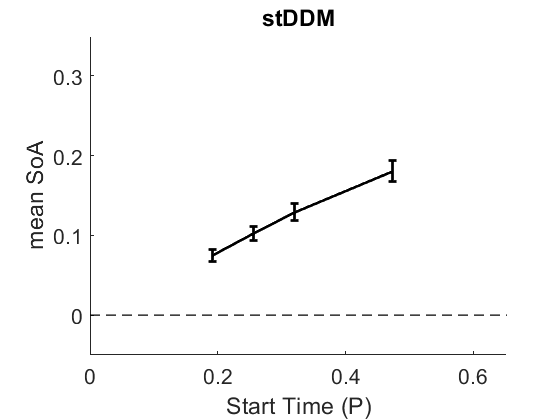

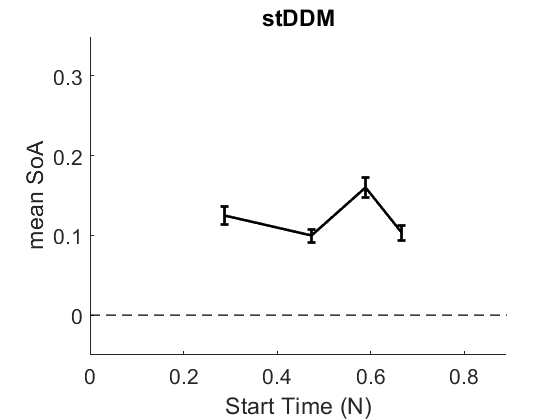

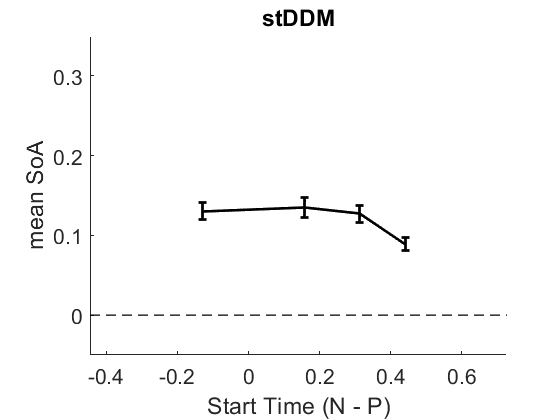


**Figure S1: Parameter relationships with spreading of alternatives.** Error bars represent 95% confidence intervals. Data was binned based on quantiles separately for each parameter and model. Data simulated based on participants with outlier values for any parameter under a given model were excluded from all plots for that model.
